# Contrasting negative stereotypes against aging through a silent disco event: an ecological study

**DOI:** 10.1007/s40520-025-03224-z

**Published:** 2025-11-14

**Authors:** Giulia Arenare, Francesco Giaquinto, Grazia Macchieraldo, Sara Bottiroli, Elena Cavallini

**Affiliations:** 1 Department of Brain and Behavioral Sciences, University of Pavia, Pavia, Italy; 2 Department of Human and Social Sciences, University of Salento, Lecce, Italy; 3 Cooperativa Onlus Piccolo Principe, Milan, Italy; 4 IRCCS Mondino Foundation, Pavia, Italy

**Keywords:** Ageism, Stigma, Intergenerational intervention, Social inclusion, Dementia

## Abstract

**Background:**

Ageism and stigma toward individuals with dementia contribute to their social exclusion and negatively impact their well-being. Intergenerational interventions, particularly those based on shared enjoyable experiences, have shown potential in reducing negative stereotypes. This study explored whether a Silent Disco event could challenge ageism and dementia-related stigma by fostering positive intergroup contact.

**Aims:**

The study assessed the effectiveness of an intergenerational Silent Disco event in reducing ageism and stigma among young adults (21–40 years, *M* = 32.76 ± 4.70), middle-aged adults (41–60, years *M* = 51.21 ± 5.88), and young-old adults (61–80 years, *M* = 66.67 ± 6.10).

**Methods:**

A 6-item questionnaire with two subscales—“Non-inclusion” and “Unpleasantness of company”—was administered to 115 participants before and after the event to measure negative stereotypes about aging and dementia. Changes in attitudes were analyzed across age groups.

**Results:**

The event reduced stigma-related unpleasantness in the total sample (*p* = .020) and ageism-related unpleasantness among middle-aged participants (*p* = .011). However, no statistically significant changes were observed in non-inclusion perceptions for either ageism or stigma.

**Discussion:**

These findings indicate that intergenerational Silent Disco events help reduce specific negative stereotypes—particularly unpleasant perceptions of older adults and people with dementia—with the strongest reduction in ageism-related unpleasantness seen in middle-aged participants. However, the persistence of non-inclusion attitudes highlights the need for broader interventions to address social exclusion.

**Conclusions:**

Silent Disco events show potential in challenging stereotypes and fostering inclusivity, but additional strategies are needed to promote deeper intergenerational engagement and social inclusion.

## Introduction

The aging population is growing significantly, doubling from 382 million in 1980 to 962 million in 2017 [1]. Although people might have a nuanced view of aging as having both positive and negative attributes, they act consciously and unconsciously to differentiate themselves from this age group due to the threats embedded in it [2]. Aging is frequently seen as a challenging phase marked by a loss of confidence and productivity [3]. Therefore, even with the increasing number of older individuals, aging is still perceived as a period of overall decline, perpetuating stereotypes that heighten the risk of prejudice and discrimination against older adults.

Prejudice and discrimination have been described as components of categorization reactions, where stereotypes represent the cognitive aspect, prejudice the affective, and discrimination the behavioral aspect of attitudes toward perceived outgroups [4]. Social categorization processes often lead individuals to overestimate differences with outgroup members and accentuate similarities with their ingroup [5]. Consequently, negative stereotypes about aging, also driven by concerns about one’s future self [6], exacerbate ageism and stigma, which can limit older adults’ participation and contribute to their social exclusion [2]. This exclusion, rooted in unfavorable stereotypes, acts as a chronic stressor that compromises older adults’ health [3].

While extensive research shows that negative stereotypes about aging are prevalent globally, evidence also supports the efficacy of interventions aimed at reducing them [7]. The aim of the present study was to assess the efficacy of an intergenerational Silent Disco event that has the potential to affect stereotypes against aging. The study explores innovative ways to mitigate ageism and stigma defined as stereotypes against older people [2].

Ageism, first introduced by Robert Butler, refers to stereotyping, prejudice and discrimination against people because of their age [6]. Stereotypes originate from the comparison among a group with actual privileges (adults), a group that is entering the privileged turn (young people), and a group that has come out of the privilege (older people) [4]. Experiencing ageism has been shown to be associated with poor mental health among older adults [8] and it is increasingly identified as a risk factor associated with anxiety, depression, and lowered life satisfaction [3]. Therefore, ageism is recognized as a public health issue and as one of the most prevalent forms of stereotyping [9]. Furthermore, as age is the major risk factor in developing dementia, older people with cognitive decline often experience dementia stigma in addition to ageist discrimination.

Stigma was initially described by Erving Goffman in 1963, and it was identified as any characteristic or attribute by which a person was devalued, tainted, or considered shameful or discredited. Subsequently, the concept of stigma has been explored in many contexts and cultures [10]. Stigma is now defined as stereotypes, prejudice and discriminatory behaviors held by people towards a person or group with a stigmatized condition [11]. Stigma is also associated with several conditions in medicine, indeed several studies have described the impact of stigmatization on patients with mental illnesses, psoriasis, or after a stroke. Stigmatization is a common phenomenon in the modern world, and it is increasingly affecting people with dementia [12].

Previous studies have indicated that younger people tend to report more ageist attitudes [13] but other studies suggest that middle-aged participants display significantly higher levels of ageism compared to both younger and older groups, particularly regarding the perceived contributions of older adults to society [14]. However, a recent study found that older adults aged 81–98 reported higher avoidant attitudes and stereotypical perceptions in comparison with older adults aged 68–73 [2].

Furthermore, ageism and stigma are not only attitudes and behaviors that an ingroup acts against an outgroup, but can be turned on themselves, implying an identity transformation resulting from individual and sociocultural influence [15]. Older people with mental health problems may hold both ageist negative attitudes toward themselves (self-ageism), as well as negative attitudes toward mental illness (self-stigma), a phenomenon called self-double stigmatization [2].

Considering these significant impacts on mental health and overall well-being, it is crucial to intervene and address ageism effectively. Reducing ageism and stigma can improve the quality of life of older adults, promote healthier aging, and create more inclusive and supportive communities. Burnes et al., [7] in a recent systematic review and meta-analysis categorized interventions to contrast ageism into intergenerational, educational, and a combination of both types. The interventions demonstrated a significant effect on improving attitudes, knowledge, and comfort towards older adults. The combined and intergenerational contact–only intervention types demonstrated to be the strongest ones in influencing attitudes and knowledge, respectively [7]. Similarly, Apriceno and Levy [16], in their systematic review and meta-analysis based on 152 studies of programs aimed at reducing ageism toward older adults, found that interventions are more effective when they combine educational components with positive intergenerational contact.

Bacsu et al. [17] used Corrigan and Penn’s stigma reduction framework (1999) to classify interventions into three categories: education (to dispel myths with facts and accurate information), contact (to provide interaction with persons with dementia), and protest (publicizing and speaking out against instances of prejudice or discrimination to suppress negative attitudes and challenge stereotypes of dementia). In their recent review, the authors report the presence of a variety of education, contact, and mixed interventions ranging from culturally tailored films to intergenerational choirs [17]. Harris and Caporella [18] tested the effects of an intergenerational choir composed of college students, individuals with dementia or mild cognitive impairment, and family members of those with dementia. They found a decrease in social isolation for the older choir members, and for the college students: a decrease in negative attitudes, an increase in positive attitudes and themes of recognizing capabilities, expanded understanding of AD, reduced stigma, and reduced social discomfort [18].

The literature suggests that, as aging occurs in both physical and social contexts, fostering interdependence and intergenerational solidarity is crucial for combating negative aging stereotypes, loneliness, and social isolation [19]. Intergenerational interaction involves the exchange of life experiences, values, and principles among people of different ages and generations [20]. Intergenerational programs (IGPs) have shown evidence of benefiting participants by reducing loneliness and exclusion for both older adults and younger generations, improving mental health, enhancing mutual understanding, and addressing issues such as ageism, housing, and care [21]. According to the intergroup contact theory [22], positive intergroup contact reduces prejudices and discrimination among groups [23].

Creating an intervention where people of different ages and health conditions can form new groups based on shared musical choices as a Silent Disco event may enhance intergroup contact. This approach has the potential to reduce stereotypes by facilitating positive interactions and fostering connections across generational boundaries. Silent Disco has become a popular event in which, rather than having a speaker system, only the participants with headphones can hear the music (“Silent Disco”, 2015). This creates a distinct in-group and out-group, whereby only those actively participating may hear the music and experience entrainment with other participants [24]. Dancing synchronously leads to greater prosocial tendencies towards partners and enhances the willingness to help an outgroup member, both among young people and children [25].

Furthermore, music listening intervention delivered by MP3 players (e.g., iPods) and headphones can support a positive role for caregivers of people with dementia [26]. Research into the effects of Silent Disco on pro-sociality and social closeness seems to be a rich field with high research potential, also regarding negative outgroup stereotypes. Suberry and Bodner [27] argued that synchronous musical activities can blur self-other boundaries, promoting a sense of group belonging or dyadic closeness.

Recent evidence reinforces the idea that musical synchrony and coordinated movement have the potential to significantly foster social bonding and reduce age-related biases. Suberry and Bodner [28] demonstrated that engaging in synchro dance movements increases social bonding and perceived closeness, especially from younger adults toward older partners, although the effect was not significantly stronger in intergenerational versus same-age dyads. Similarly, Kim and Lao [29] found that synchronous walking led to improved sentiments from younger adults toward older adults, enhancing connectedness, self-other merging, general liking, and interpersonal impressions.

These studies highlight how shared rhythmic activities may serve as powerful vehicles for promoting intergenerational connection.

Furthermore, Silent Disco paradigms have recently been employed to isolate the effects of synchrony in musical interactions. A recent study showed that dyads dancing in synchrony rated their partner interactions more positively compared to those in asynchronous conditions, indicating that temporal alignment strengthens interpersonal connection [30]. In earlier work, the same authors observed that synchronized dancing increased subjective ratings of affiliation [31].

These findings support the use of Silent Disco not only as a novel and enjoyable approach but also as an effective way to promote social connection between different generations.

### The present study

The present study utilized a novel approach by implementing a Silent Disco event as an intergenerational intervention aimed at promoting intergroup understanding and contrasting negative stereotypes, prejudice and discrimination which can be expressed through descriptive sentences about how group members are perceived [13] or prescriptive beliefs about the allocation of social resources [32]. The main aim of the present study was to assess the efficacy of a Silent Disco approach in modifying ageism and stigma in three age groups: young, middle-aged, and young-old participants. In addition, given the potential differences in stereotypical behaviors across age cohorts we also investigated the impact of age on ageism and stigma and on the intervention effects. Hence, we included three age groups: younger, middle-aged, and young-old adults.

Based on the insights from previous research [33], we hypothesized that participation in an intergenerational Silent Disco event would effectively reduce these forms of stereotypes. Through the shared experience of music and dance, we aimed to promote more positive attitudes towards aging and aging with different health conditions. This initiative was grounded in Allport’s [22] intergroup contact theory, which posits that positive interactions between different groups can reduce prejudices. By providing a shared, enjoyable experience, the Silent Disco aimed to foster better understanding and strengthen social contact across generations.

## Methods

### Participants

The participants were people who spontaneously decided to attend the event. Advertising was carried out through social networks, posters, and inserts in local newspapers. The announcement presented the event with a charitable purpose, and as an opportunity to meet people of different ages and health statuses to raise awareness of the dementia issue. The information collected concerned gender, age, minutes of stay at the event, companionship (with someone with the same age or different age), motivation (charity or other reasons) and the level of education (from “1 = primary school diploma” to “5 = post-degree specialization”). One hundred forty-seven participants completed the brief self-report questionnaire and 115 over 20 and under 80 years old were selected for minutes of staying (> 30 min) between 5.00 pm and 7.00 pm, the time slot with the highest attendance and the best proportion among groups of different age ranges. The final sample was composed by 73 females (63%), and 42 males (37%) with a mean age of 50.05 (*SD* = 14.07). Participants spent an average of 118.1 min at the event (*SD* = 38.80). The majority had a high level of education, with approximately 67% holding a degree or post degree title. The sample was divided into three subgroups based on age: young adults (20–40 y.o; *M* = 32.76 ± 4.70.), middle-aged adults (41–60 y.o *M* = 51.21 ± 5.88), and young-old adults (61–80 y.o. *M* = 66.67 ± 6.09) (Table 1). Partial data from a post-event questionnaire, completed by only a subset of the sample, indicated that seven participants stated that they were attending the event as informal caregivers of older adults with dementia. However, due to the nature of the event, it was not possible to gather further details regarding the type of dementia.

**Table 1:** Descriptive analysis of characteristics of the sample for age groups

Characteristic	Young adults (*n = 34*)	Middle-aged adults (*n = 49*)	Young-old adults (*n = 32*)	*p*
*Sex (F*,* %)* ^*a*^	20 (59%)	30 (61%)	23 (72%)	0.497 ^b^
*Education (n*,* %)* ^*a*^				0.001^b^
Primary	0 (0%)	0 (0%)	1 (3%)	
Lower secondary	0 (0%)	1 (2%)	3 (9%)	
Upper secondary	3 (9%)	21 (43%)	9 (28%)	
Degree	23 (68%)	20 (41%)	19 (59%)	
Post degree	8 (24%)	7 (14%)	0 (0%)	
*Age*	32.76 ± 4.70	51.21 ± 5.88	66.67 ± 6.10	
*Time spent*	115.5 ± 43.84	114.1 ± 30.61	127.2 ± 43.82	0.299

Values are means ± standard deviations, unless specified otherwise. F: Females, M: Males,^a^ Proportions^b^ Chi-square test

### Measures

We developed a 6-items questionnaire for a fast application at the entry and the exit of the event, to capture the instinctive response about six negative stereotypical sentences concerning older adults and people with dementia. Respondents expressed their level of agreement with the statements using a 6-points scale (from 1 = “I totally disagree” to 6 = “I totally agree”). Higher scores on the scale indicate greater endorsement of the negative stereotypes, meaning that a higher score reflects greater levels of ageism or stigma. We have extracted three items from two validated tools largely used to assess ageism: the Fraboni Scale of Ageism [13] and the Intergenerational-Tension Ageism Scale [32].

The Fraboni Scale of Ageism is a 29-item self-report scale, developed by Fraboni, Saltatone and Hughes [13], to provide a multidimensional assessment of ageism, considering cognitive, affective, and behavioral dimensions.

The item “*the company of most older people is quite enjoyable*” used in our questionnaire belongs to the dimension “discrimination” in the original form [13], and to “negative emotions and discrimination” in the Italian validation [34].

The Intergenerational-Tension Ageism Scale [32] focuses on 20 prescriptive beliefs, using sentences with a “should-based” form, concerning potential intergenerational tensions: “succession of envied resource”, derives from expectations surrounding enviable resources and societal positions; “symbolic *identity* avoidance”, focuses on passive depletion of currently shared resources; and “consumption of shared-resource”, involves resources more symbolic than succession or consumption, limiting elder participation in activities usually reserved for younger people.

The two items used in our questionnaire, “*older people typically shouldn’t go to places where younger people hang out*”, and “*generally older people shouldn’t go clubbing*”, derive from the third dimension, and were translated in Italian by an English and Italian native language.

To measure the stigma against people with dementia, other three items were developed changing the words “older people” with “people with dementia”. Cronbach’s alpha for the “ageism score” including all three items was 0.451 (95% CI: 0.274–0.592), and for the “negative stereotypes against people with dementia score” was 0.633 (95% CI: 0.505–0.732). Referring to the Cronbach’s alpha value it was decided to partition the items according to their original scales. As a result, two subgroups for each scale emerge: for the ageism scale, items 2 and 3 paired together as the “Non-inclusion Ageism” with a Cronbach’s alpha of 0.697 (95% CI: 0.565–0.793). While item 1 alone will be labelled as the “Unpleasantness of company Ageism”; for the negative stereotypes against people with dementia scale, items 2 and 3 paired together as the “Non-inclusion Stigma” with a Cronbach’s alpha of 0.796 (95% CI: 0.707–0.860). While item 1 alone will be labelled as the “Unpleasantness of company Stigma”. The original scales showed low internal consistency, and although dividing the items into subscales improved reliability, the overall findings will be considered exploratory.

### Procedures

The participants were asked to complete the brief questionnaire twice: at the entrance and at the exit of the event. Each subject has been assigned a code to safeguard anonymity and compare the pre and post responses of each individual subject. Two assistants oversaw the correct compilation of the questionnaires and the application of the correct code.

During the intervention, participants were exposed to three distinct playlists delivered through color-coded headphones. The playlists consisted of: (1) 1950s–60s music, featuring boogie-woogie, mazurka, and rock and roll genres (blue headphones); (2) 1970s–80s music, featuring dance and rock genres (red headphones); and (3) 1990–2000 s music, featuring ska, reggae, and commercial dance genres (green headphones). The selection aimed to cover a broad range of musical eras and style to accommodate participants’ potential preferences and promote engagement across different age groups. Participants were free to choose their preferred channel and could switch between playlists at any time during the event.

### Informant consent

All the participants gave written consent for participation in the research. The purpose of the event, research manager, phases of the research, and the possibility of renouncing to participate without compromising the continuation of the study, were explicated. Data processing has been ensured to be strictly confidential and anonymous for purposes of research, complying with the right to privacy protection. According to the Declaration of Helsinki, informed consent from the legally authorized representative was obtained for the mentally incapables. The study was approved by the Ethical Committee of the Department of Brain and Behavioural Sciences of the University of Pavia (n. 091/21).

### Statistical analysis

After stratifying the participants into three age groups (Young adults, Middle-aged adults, Young-old adults), all demographic and questionnaire variables were compared between the groups using ANOVA. The chi-square test was used to examine differences in gender, education, companion status, and motivation. For the two subscales of Unpleasantness was used the reversed score, a minor score represents a minor Unpleasantness. Differences between the Non-inclusion and Unpleasantness scales in ageism and stigma were investigated using t-tests. ANOVA was used to investigate whether the initial scores of ageism and stigma could influence the scores after the event. Repeated measures ANOVA was employed to investigate the pre and post differences between the 4 measures of ageism and stigma with age and education included as covariates. A two-tailed significance threshold value of *p* < .05 was used for all models. The RStudio software (Version 1.3.1056, Boston, MA, USA) was used for all statistical analyses.

## Results

### Baseline

No significant differences among age groups were found about Ageism Unpleasantness (F_2, 112_ = 0.388, *p* = .679) and Stigma Unpleasantness (F_2,112_ = 1.544, *p* = .218) but significant differences in perception across different age groups were found about Ageism Non-inclusion (F_2,112_ = 3.934, *p* = .022) and Stigma Non-Inclusion (F_2,112_ = 4.586, *p* = .012) (Tables 2 and 3). The post-hoc analysis was performed using the Tukey test, the p value < 0.05 was considered to be statistically significant. For the Ageism Non-inclusion, young-old adults had higher scores compared to young adults. However, no significant differences were found between young-old adults and the middle-aged group or between young adults and the middle-aged group. Similarly, for the Stigma Non-inclusion, young-old adults had significantly higher scores than young adults, but no significant differences were observed between young-old adults and the middle-aged group or between young adults and the middle-aged group. Considering these baseline differences, an ANOVA was conducted, using education and age as covariates, to investigate whether the initial scores of Ageism and Stigma Non-Inclusion could influence the scores after the event. It was found that for Stigma Non-Inclusion post event, education was not a significant predictor (F _1,110_ = 0.005, *p =* .943) as Age group (F _2,110_ = 2.41, *p* = .095), while initial Stigma Non-Inclusion scores significantly influenced the post-event scores (F _1,110_ = 56.69, *p* < .001). For Ageism Non-Inclusion post event, age group did not significantly predict the post-event scores (F _2,110_ = 0.67, *p* = .515) as education (F _1,110_ = 0.085, *p* = .772), while initial Ageism Non-Inclusion scores were a significant predictor (F _1,110_ = 46.53, *p* < .001).

We subsequently analyzed the differences in Stigma and Ageism across the two subscales (Non-inclusion Vs. Unpleasantness) to determine if any significant discrepancies existed. Our findings revealed that individuals reported higher levels of Non-inclusion compared to Unpleasantness for the Stigma (F_1,111_ = 7.32, *p* = .008) but not for the Ageism (F_1,111_ = 3.915, *p* = .050), even if it is marginally significant (Table 2).

**Table 2:** Descriptive analysis of pre-test measures for age groups

Variable	Young adults	Middle-aged adults	Young-old adults	*p*
Ageism Non-Inclusion	4.08 ± 1.75	4.42 ± 2.26	5.68 ± 3.22	0.022^#^
Unpleasantness	2.47 ± 0.96	2.54 ± 0.87	2.34 ± 1.15	0.679
*Stigma*				
Non-Inclusion	3.62 ± 1.84^a < c^	4.62 ± 2.51	5.56 ± 3.33	**0.012**
Unpleasantness	2.91 ± 0.97	3.31 ± 1.16	3.35 ± 1.33	0.218
*Companion* ^a^				**0.034** ^b^
Same age	29 (85.29%)	30 (61.22%)	19 (59.38%)	
Different age	5 (14.71%)	19 (38.78%)	13 (40.63%)	
*Motivation* ^a^				0.466 ^b^
Charity	14 (41.17%)	23 (46.94%)	18 (56.25%)	
Other reasons	20 (58.82%)	26 (53.06%)	14 (43.75%)	

Values are means ± standard deviations, unless specified otherwise. ^a^ Proportions ^b^ Chi-square test. # = Young adults < Young-old adults

### Silent disco effects

To test the impact of the intergenerational Silent Disco event on participants negative stereotypes, a repeated measures ANOVAs were run on the four measures of stigma and ageism, using age groups as between subjects factor, and education as covariate variable. The analysis revealed a significant main effect of the Silent Disco event on Stigma Unpleasantness (F₁,₁₁₁ = 5.575, *p* = .020), with education also showing a significant effect (F₁,₁₁₁ = 4.125, *p* = .045), while age group did not significantly influence this measure (F₂,₁₁₁ = 1.482, *p* = .232). No significant main effects were found for Ageism Non-inclusion (Silent Disco: F₁,₁₁₁ = 1.053, *p* = .307; Age group: F₂,₁₁₁ = 0.694, *p* = .502; Education: F₁,₁₁₁ = 0.196, *p* = .659), Ageism Unpleasantness (Silent Disco: F₁,₁₁₁ = 0.393, *p* = .532; Education: F₁,₁₁₁ = 0.050, *p* = .824), or Stigma Non-inclusion (Silent Disco: F₁,₁₁₁ = 1.517, *p* = .221; Age group: F₂,₁₁₁ = 0.888, *p* = .415; Education: F₁,₁₁₁ = 0.983, *p* = .324). However, a significant interaction between Silent Disco participation and age group was found for Ageism Unpleasantness (F₂,₁₁₁ = 4.729, *p* = .011). Post hoc analysis showed that only the middle-aged group exhibited a significant reduction in unpleasantness related to ageism from pre- to post-event (mean pre = 2.54 ± 0.87; post = 1.94 ± 0.80). (Tables 3 and 4).

**Table 3:** Pre and post descriptives analysis for ageism and stigma

Variables	Young adults		Middle-aged adults		Young-old adults	
	Pre	Post	Pre	Post	Pre	Post
*Ageism*						
Non-inclusion	4.08 ± 1.75	3.85 ± 1.67	4.42 ± 2.26	3.67 ± 2.07	5.68 ± 3.22	4.69 ± 2.45
Unpleasantness	2.47 ± 0.96	2.36 ± 1.01	2.54 ± 0.87	1.94 ± 0.80	2.34 ± 1.15	2.10 ± 0.78
*Stigma*						
Non-inclusion	3.62 ± 1.84	3.56 ± 1.81	4.62 ± 2.51	3.92 ± 2.20	5.56 ± 3.33	5.41 ± 3.16
Unpleasantness	2.91 ± 0.97	2.80 ± 1.01	3.31 ± 1.16	2.86 ± 1.22	3.35 ± 1.33	3.28 ± 1.06

Values are means ± standard deviations

**Table 4:** Repeated measures ANOVA

Questionnaires	Silent Disco	Silent Disco for Age	Education
Ageism Non-inclusion	F_1,111_ = 1.053; *p =* .307	F_2,111_ = 0.694; *p* = .502	F_1,111_ = 0.196; *p* = .659
Ageism Unpleasantness	F_1,111_ = 0.393; *p* = .532	F_2,111_ = 4.729; *p* = **.011**	F_1,111_ = 0.050; *p* = .824
Stigma Non-inclusion	F_1,111_ = 1.517; *p* = .221	F_2,111_ = 0.888; *p* = .415	F_1,111_ = 0.983; *p* = .324
Stigma Unpleasantness	F_1,111_ = 5.575; *p* = **.020**	F_2,111_ = 1.482; *p* = .232	F_1,111_ = 4.125; *p* = .045

Repeated measures ANOVA using age groups as between subjects’ factor, and education as covariate variable.

## Discussion

The primary focus of this study was to evaluate the effects of an intergenerational Silent Disco event on ageism and stigma across three age groups. The most notable findings pertain to changes in negative stereotypes related to the unpleasantness of the company of old people and individuals with dementia. This reduction indicates that the Silent Disco event had a positive impact, revealing that participants across the three age groups found the company of individuals with dementia more enjoyable after the event. This aligns with studies showing that greater contact with people with dementia is associated with changes in attitudes [35] and a lower level of stigma [36]. Interestingly, both studies [35, 36] focused on care-workers and people not working with older-adults affected by dementia and found that care-workers reported higher more positive attitudes towards dementia respect to people not working with people affected by dementia. Even though only a small number of participants were people with dementia, and no structured or explicit interaction with individuals with dementia was planned, it remains plausible that indirect forms of intergroup contact contributed to the observed reduction in stigma. According to recent developments in intergroup contact theory, indirect contact mechanisms—such as imagined contact, vicarious contact, or parasocial contact—can also promote positive attitude change in the absence of direct interpersonal interaction [37, 38]. These forms of contact are especially relevant in real-world contexts where the stigmatized outgroup is either invisible or underrepresented, as in the case of dementia, a condition often concealed due to stigma or communication difficulties. In our study, the awareness of the theme, highlighted in the promotional material and reinforced during the event, may have activated such mechanisms.

However, not only working with older people affected by dementia can change the perception of the disease, but also engaging in intergenerational enjoyable activities can help shift these perspectives. Indeed, a recent study [18] assessed the effect of an intergenerational choir on stigma and found that college students reduced negative attitudes and stigma. Through this social contact, they were able to show the students a different perspective about dementia and demonstrated that people with dementia can be enjoyable, despite the challenges of the disease.

In our study, the Silent Disco event may have functioned similarly, that is providing enjoyable contact has determined a decreasing in discriminatory behavior by demonstrating that people with dementia can be pleasant company in various settings.

These findings suggest that contact opportunities can reduce stigma and have implications for the development of interventions. Regarding Ageism Unpleasantness, it is interesting to note that we found an interaction effect between age and Silent Disco. Specifically, the middle-aged group showed a significant increasing in pleasantness of the company of older people.

This result is very interesting when considering that the change occurred specifically in the age group that already spends more time with older people by default. Indeed, even at the silent disco, middle-aged adults attended alongside older people, while young people went to the event with their peers. This shift in attitude highlights the value of the silent disco, emphasizing that it is not enough just to be with older people to appreciate their company; it is necessary to create and, therefore, share enjoyable moments/events which is consistent with Allport’s [22]intergroup contact theory.

Regarding the Silent Disco event, it is interesting to analyze the possible mechanisms responsible for its effectiveness. In our study, participants were free to choose and switch among the three playlists delivered through color-coded headphones, which means that not all individuals were exposed to the same musical content. This variability may has contributed in a different way to embodied experiences. Notably, people deciding to switch to the same headphone color were likely dancing to the same rhythm and tempo, possibly fostering spontaneous synchrony in movement. Although not directly measured, such synchrony has been associated in prior research with increased social bonding, positive affect, and self–other merging [27–31]. At the same time, all participants were immersed in a shared social context likely characterized by mutual smiling, eye contact, and the collective energy typical of group dance settings, which may have further enhanced a sense of connection. These dynamics suggest that musical synchrony and shared affective experiences may represent meaningful mechanisms of change underlying the observed reductions in stigma and age-related unpleasantness.

Another aspect that seemed useful to analyze, beside of the silent disco, is the comparison between the subscales of ageism and stigma at baseline. These data can provide useful insights into the type of stigma present in the population. We found that the Stigma Non-inclusion subscale has a higher score than the Stigma Unpleasantness. The items used in the Non-inclusion subscale are part of the “identity-based prescriptions”, which are beliefs that older individuals with dementia should not infringe on symbolic resources by engaging in activities and roles that are usually reserved for the young (e.g., go to places for younger people, such as discotheque).

In contemporary society, increasingly segregated by stereotypes, there are limited opportunities for positive and meaningful interactions between people with different ages and conditions [39]. These initial findings may explain why we observed a significant reduction only in the Unpleasantness scale. Interventions like our Intergenerational Silent Disco can help reduce this segregation and demonstrate that people of different ages and conditions can enjoy each other’s company.

However, altering deeply rooted beliefs regarding who is permitted to occupy certain spaces remains a significant challenge. These findings underscore the need for public health interventions to implement psycho-social strategies to strengthen social support.

## Conclusion

Although an increasing number of studies have investigated the role of different interventions to reduce negative stereotypes about aging, many types of interventions remain to be explored. Greater attention to this crucial issue is needed, as it has significant implications for society.

Our intervention results more effectively in reducing negative stereotypes about the unpleasantness of the company of older people and people with dementia. Despite the promising findings, several limitations should be acknowledged. Notably, the original scales exhibited low internal consistency, and although subscales improved reliability, results remain exploratory. Given the study’s reliance on the subjectivity of self-report measures, other observational instruments should be used, such as video recording. Furthermore, the lack of a control group can restrict the generalizability of the results. An additional limitation concerns the small number of older adults with dementia attending the event. Nevertheless, we believe that this number was sufficient to contribute, at least in part, to reducing the stigma against older adults.

However, this study offers the significant advantage of being an ecological study and insights to continue developing and implementing such interventions.

In summary, intergenerational Silent Disco approach not only provides a tangible opportunity for contact among young people, middle-aged people, young-old adults, and individuals with dementia but also create an inclusive and enjoyable environment. Future research could employ longitudinal designs to examine the long-term effects of intergenerational interventions on attitudes towards aging and dementia and use specific measures for self-ageism and self-stigma.

Additionally, exploring the underlying mechanisms driving stigma and ageism reduction, such as increased social interaction and empathy, would provide valuable insights. Among these mechanisms, shared musical experiences and the possibility of spontaneous synchronization may play a meaningful role in enhancing intergroup closeness and should be examined more directly in future studies. The reduction of stigma is important to remove barriers to early diagnosis, intervention, and community support.

## Data Availability

The dataset used and analyzed during the current study is available from the corresponding author on request.
